# The Care of the Baby

**Published:** 1895-12

**Authors:** 


THE CARE OF THE BABY. A Manual for Mothers and Nurses, Combining Practical Directions for the Management of Infancy and Childhood in Health and in Disease. By J. P. Crozier Griffith, M. D., Clinical Professor of Diseases of Children in the Hospital of the University of Pennsylvania; Professor of Clinical Medicine in the Philadelphia Polyclinic. Philadelphia: W. B. Saunders, 925 Walnut Street. 1895. 8vo. Cloth, $1.50.

The plain rules laid down by Dr. Griffith in this volume, and the many practical points discussed, all in language easily understood by the laity, will make this one of the most useful books for mothers and nurses. It is a safe and reliable guide for the home management of children, first to keep them in the healthiest possible condition, and secondly, to properly care for them in sickness. Instructions are given for making the necessary preparations for the baby before its birth—then for the new-born child, its toilet, clothes, feeding, exercise, sleep, nurses, rooms, and all that concerns the health and growth of the child. An appendix contains a useful dietary, simple receipts for internal medicines, local applications, etc. In the hands of mothers and nurses it will save the physician much time in the matter of giving detailed directions for the general management of the child.

